# Quantification of the relative contribution of estrogen to bone mineral density in men and women

**DOI:** 10.1186/1471-2474-14-366

**Published:** 2013-12-23

**Authors:** Lan T Ho-Pham, Nguyen D Nguyen, Tuan V Nguyen

**Affiliations:** 1 Department of Internal Medicine, Pham Ngoc Thach University of Medicine, Thanh Thai Street, District 10, Ho Chi Minh City, Vietnam; 2 Department of Rheumatology, People’s Hospital 115, Ho Chi Minh City, Vietnam; 3 Osteoporosis and Bone Biology Program, Garvan Institute of Medical Research, Sydney, Australia; 4 St Vincent’s Clinical School, Faculty of Medicine, University of New South Wales, Sydney, Australia; 5 Bone and Muscle Research Unit, Ton Duc Thang University, Ho Chi Minh City, Vietnam

**Keywords:** Estradiol, Testosterone, Bone mineral density, Osteoporosis, Men

## Abstract

**Background:**

The study quantified the relative contributions of estrogen (E2) and total testosterone (TT) to variation in bone mineral density in men and women.

**Methods:**

This was a cross-sectional study which involved 200 men and 415 women aged 18 to 89 years. BMD at the lumbar spine (LS) and femoral neck (FN) was measured by DXA. Serum levels of E2 and TT were measured by electrochemiluminescence immunoassays. The association between E2, TT, and BMD was analyzed by the multiple linear regression model, adjusting for age and BMI. The contribution of each hormone to the variation in BMD was quantified by the bootstrap method.

**Results:**

In women, higher serum levels of E2, but not TT, were significantly associated with greater BMD at the FN (*P* = 0.001) and LS (*P* < 0.0001). In men, higher serum levels of E2 were independently associated with greater FNBMD (*P =* 0.008) and LSBMD (*P =* 0.086). In the multiple linear regression model, age, body weight and E2 accounted for 50-55% variance in FNBMD, and 25% (in men) and 48% (in women) variance in LSBMD. Variation in E2 accounted for 2.5% (95% CI 0.4 - 7.8%) and 11.3% (95% CI 8.1 - 15.3%) variation in FNBMD in men and women, respectively. Moreover, E2 contributed 1.2% (95% CI 0.1 - 5.8%) and 11.7% (95% CI 8.5 - 15.9%) variation in LSBMD in men and women, respectively.

**Conclusions:**

Estrogen is more important than testosterone in the determination of age-related bone mineral density men and women of Vietnamese background. However, the relative contributions of estrogen to bone mineral density in men are likely modest.

## Background

That estrogen plays a critical role in bone health in women is a well-known fact. Deficiency of estrogen leads to increased bone loss, lower bone mineral density (BMD), and increased fracture risk [1]. In women with reduced bone density and/or an existing fracture, treatment with estrogen could reduce bone loss, and lower fracture risk [2]. Thus, the BMD-mediated causal relationship between estrogen and fracture in women has been well established.

However, recently emerging evidence has suggested that estrogen also has important effects on bone health in men. Almost 20 years ago, Smith and colleagues reported a 28-year old man with a bone age of 15 years, who had estrogen resistance due to disruptive mutation of the estrogen receptor gene [3]. The man had normal level of testosterone and elevated estradiol, but had severe osteopenia associated with increased values of bone turnover markers. Subsequently, two young men with undetectable estradiol levels due to mutation in the CYP19A1 gene (this gene is responsible for converting androgens to estrogens) were identified [4,5]. The two men also had low bone density as initially reported by Smith et al.; however, when the men were treated with estrogen, their BMD increased. These cases have demonstrated that estrogen does play an important role in the skeletal maturation and mineralization in men, and changes the traditional view that estrogen is a “female hormone”.

A series of subsequent case reports have indicated that estrogen is essential for the normal growth and maturation of the skeleton in men with aromatase deficiency and low serum estradiol (E2) levels [4-7]. Some cross-sectional studies have further demonstrated a significant positive relationship between serum E levels and BMD in men [8-14]. Most of these studies were based on elderly populations of Caucasian background, and as a result, it is difficult to assess the relative contribution of estrogen to the inter-individual variation in BMD in the general population.

Although these studies collectively suggest that estrogen might be an important factor for bone growth and bone maintenance in men, most studies have not considered the simultaneous effects of estrogen and testosterone on BMD in men. The question of interest is therefore: what is the proportion of variation in BMD among men and women of Asian background that can be attributed to the variations in estrogen and testosterone. The present study was designed to address that research question by analyzing the relative contributions of serum levels of estradiol and testosterone to the variation in BMD in Asian men and women.

## Methods

### Study design and participants

The study was designed as a cross-sectional investigation, with the setting being Ho Chi Minh City, a major city in Vietnam. The research protocol and procedures were approved by the Scientific Committee of the People’s Hospital 115 and Pham Ngoc Thach University of Medicine. All volunteer participants were provided with full information about the study’s procedures and aims, and gave informed consent to participate in the study, according to the principles of medical ethics of the World Health Organization and the Helsinki Declaration.

We used simple random sampling technique to identify potential participants. We approached community organizations, including churches and temples, and obtained the list of members, and then randomly selected individuals aged 18 or above. We sent a letter of invitation to the selected individuals. The participants received a free health check-up, and results of lipid analysis, but did not receive any financial incentive. Participants were excluded from the study if they had diseases deemed to affect to bone metabolism, such as hyperthyroidism, hyperparathyroidism, chronic kidney disease, malabsorption syndrome, alcoholism, chronic colitis, multiple myeloma, leukemia or chronic arthritis.

### Measurements and data collection

Data collection was done by research doctors and nurses using a validated questionnaire. The questionnaire solicites the following information from participants: anthropometric factors, lifestyle factors, dietary intakes, physical activity, and clinical history. Anthropometric parameters including age, weight, standing height were obtained. Age was calculated from the date of birth to the date of interview. Height without shoes (in centimeters) was measured to the nearest 0.1 cm by a wall-mounted stadiometer. Weight, without shoes or clothing, was measured (to the nearest 0.1 kg) on an electronic scale. Body mass index (BMI) was then derived as the ratio of weight (kg) over height squared (in m^2^).

### Bone mineral density

Areal BMD was measured at the lumbar spine (L2-L4), femoral neck, and whole body using a Hologic QDR 4500 (Hologic Corp, Madison, WI, USA). The short-term in vivo expressed as the coefficient of variation was 1.8% for the lumbar spine and 1.5% for the hip. The machine was standardized by a standard phantom before each measurement. BMD at femoral neck was used as a main variable for analysis.

### Measurement of sex hormones

Blood samples were collected between 8 AM and 10 AM after an overnight fast. Serum concentrations of total testosterone (TT) and estradiol (E2) were measured by electrochemiluminescence immunoassay (ECLIA) on an Roche Elecsys 10100/201 system (Roche Diagnosis Elecsys). This method can measure the concentration of estradiol in the range of 5–4,300 pg/ml (18.4-15781 pmol/L), and total testosterone in the range of 25–15,000 pg/ml (87–52000 pmol/L). The sensitivity of the assay is 2.18 pg/ml with an intraassay CV of 6.2% at 34.8 pg/ml and 2.8% at 1018 pg/ml. The inter-assay CV at these two levels was <20%.

### Data analysis

The association between TT, E2, and BMD was analyzed by the multiple linear regression model, with BMD being the outcome variable, and TT and E2 predictor variables. Because both BMD and concentration of E2 or TT change with age and weight, the analysis was adjusted for the effects of age and body mass index (BMI). Because the distribution of E2 and TT was not normally distributed, we transformed the variables into natural logarithmic scale before analysis. The statistical significance of each predictor variable was assessed by the t-test after adjusting for all other variables in the model. A nominal P value of 0.05 was considered “statistical significance”. The coefficient of determination (R^2^) from the most optimal model was obtained and further analyzed. In order to address the relative importance of each and combined predictors, we used the “lmg” method [15] to decompose the overall R^2^ into individual effect. The R program was used for the statistical analysis [16] and the package relaimpo within R package [17] was used to estimate the relative contribution of individual predictor variable.

## Results

The study involved 205 men and 432 women, aged between 18 and 87 years (Table 1). The average age among men was 44, which was slightly lower than that among women (48 years). Fifty-one percent of men and 0.7% of women reported being current smokers. Approximately 20% men and 13% women were obese (body mass index greater than 25 kg/m^2^). On average, men had significantly lower E2 and higher TT concentrations than women. The mean value of TT was 487 pg/mL in men and 20 pg/mL in women; and that of E2 was 28 pg/mL and 80 pg/mL in men and women, respectively.

**Table 1:** Characteristics of 205 men and 432 women

**Variable**	**Men**	**Women**	**P-value**
N	205	432	
Age (yr)	43.8 (18.4)	47.7 (17.1)	0.009
Weight (kg)	61.1 (9.2)	52.2 (7.6)	<0.0001
Height (cm)	164.2 (6.6)	153.4 (5.3)	<0.0001
Body mass index (kg/m^2^)	22.7 (3.2)	22.2 (3.0)	0.091
Current smoking	105 (51%)	3 (0.7%)	<0.0001
Testosterone (pg/mL)	487 (208)	20 (32)	<0.0001
Estradiol (pg/mL)	28 (42)	80 (237)	<0.0001

Note: Data are shown in mean and standard deviation (in bracket).

### Sex hormones and age

**Figure 1:**
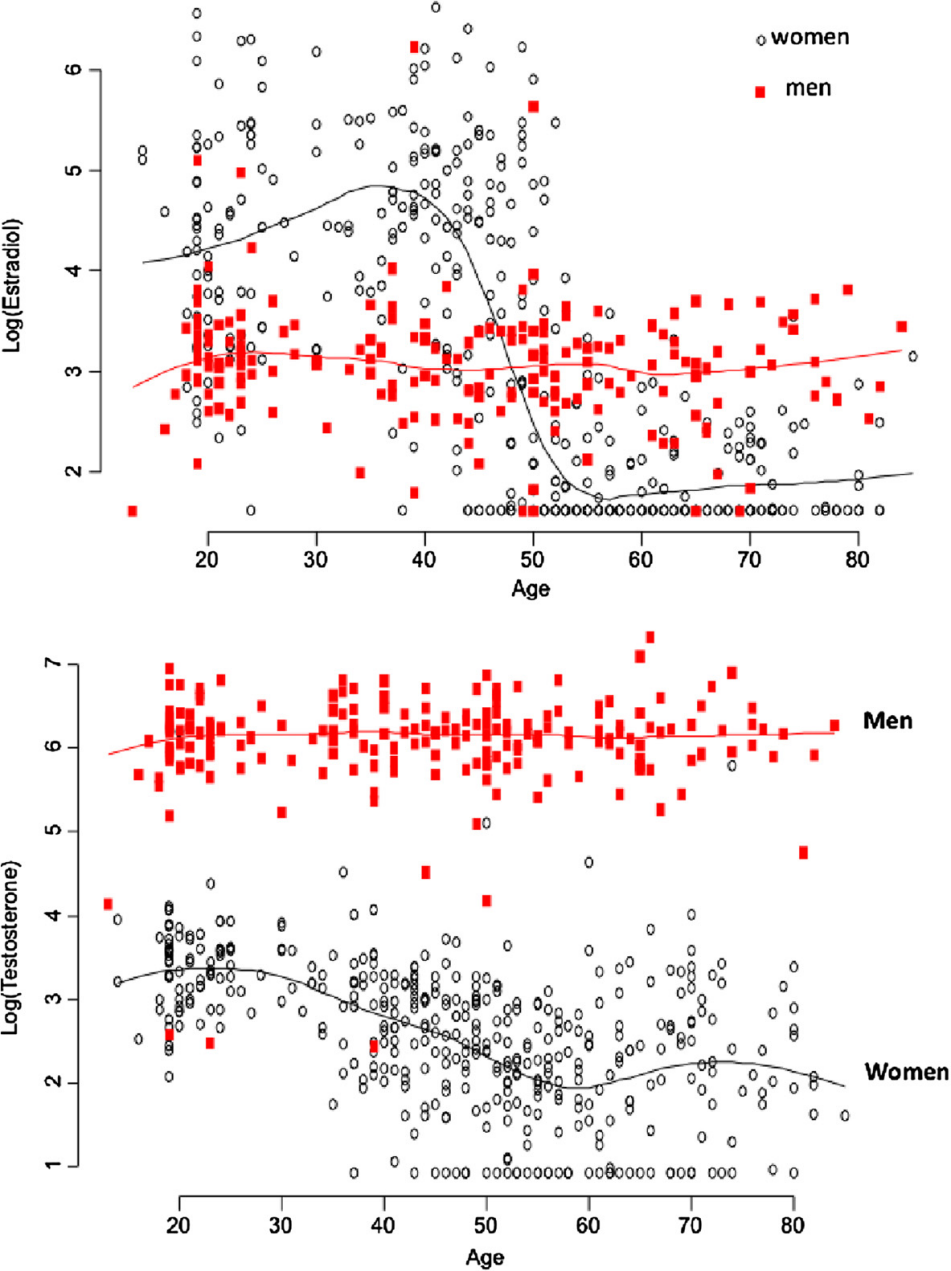
Association between estradiol (upper panel) and testosterone (below panel) with age in men (red) and women (black).

The relationship between E2 and age in women was described by the third-degree polynomial regression model (Figure 1). As expected, serum levels of estradiol remained stable between the ages of 18 and 35, followed by a rapid decrease between the age of 40 and 60. There was no noticeable change in estradiol between the age of 60 and 70 years. In contrast, the serum levels of estradiol in men declined gradually with advancing age, but the magnitude of decline was modest, with 0.8% per year.

The relationship between age and total testosterone was also different between sexes. In men, there was no appreciable change in TT with advancing age. However, in women, there was a linear decline of total testosterone levels with advancing age, with the rate of decrease being 2.5% per year.

### Sex hormones and BMD

**Figure 2:**
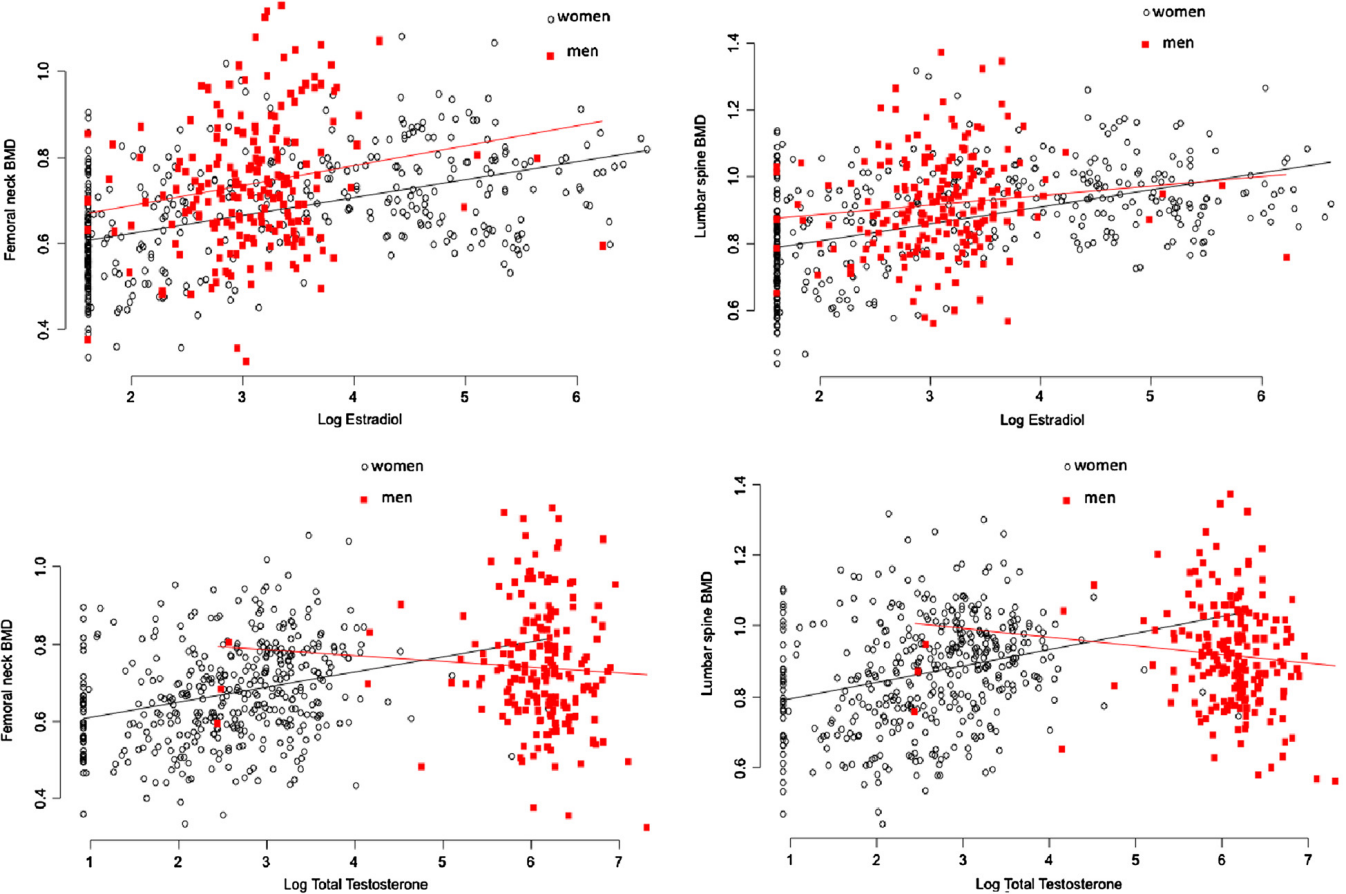
Association between estradiol (upper panel) and testosterone (lower panel) and BMD in men (red) and women (black), at the femoral neck (left) and lumbar spine (right).

In univariate analysis, individuals with higher serum levels of E2 also had higher BMD at the femoral neck and lumbar spine in both men and women (Figure 2). There was no appreciable association between total testosterone and BMD in men, but a positive relationship was observed in women.

For a given age and body weight, men with greater serum levels of estradiol had greater BMD at the femoral neck and lumbar spine (Table 2). In the presence of estradiol, total testosterone was not a significant predictor of BMD in men. In women, estradiol but not total testosterone was a significant predictor of BMD. The magnitude of association between estradiol and BMD in men was comparable with (in the case of lumbar spine BMD) or greater than that (femoral neck BMD) in women. Each standard deviation lower in log estradiol was associated with 0.032 g/cm^2^ lower in femoral neck BMD in men; the estimate for women was 0.014 g/cm^2^.

**Table 2:** Association between sex hormones and bone mineral density: the linear regression model

**Variable**	**Men**		**Women**	
	**Coeffcient (SE)**	**P-value**	**Coeffcient (SE)**	**P-value**
**Femoral neck BMD**				
Intercept	0.097 (0.105)	0.354	0.443 (0.032)	<0.0001
Age	-1.152 (0.104)	<0.0001	-1.483 (0.121)	<0.0001
Age squared	0.023 (0.101)	0.820	-0.268 (0.096)	0.005
Weight	0.07 (0.0008)	<0.0001	0.004 (0.0008)	<0.0001
Log (testosterone)	0.020 (0.012)	0.090	-0.004 (0.0005)	0.530
**Log (estradiol)**	**0.032 (0.120)**	**0.008**	**0.014 (0.004)**	**0.001**
**Lumbar spine BMD**				
Intercept	0.411 (0.131)	0.002	0.554 (0.040)	<0.0001
Age	-0.293 (0.130)	0.025	-1.360 (0.150)	<0.0001
Age squared	-0.253 (0.127)	0.048	-0.765 (0.119)	<0.0001
Weight	0.007 (0.001)	<0.0001	0.004 (0.0006)	<0.0001
Log (testosterone)	-0.001 (0.015)	0.951	0.006 (0.008)	0.401
**Log (estradiol)**	**0.026 (0.015)**	**0.086**	**0.021 (0.005)**	**<0.0001**

Note: SE, standard error.

### Analysis of relative importance

Relative importance analysis (Table 3) shows that variation in serum levels of estradiol “explained” 2.5% (95% CI 0.4 to 7.8%) of variance in femoral neck BMD. However, in women, the relative contribution of estradiol to BMD variation was even greater: 11.3% (95% CI 8.1 to 15.3%) for femoral neck BMD and 11.7% (95% CI 8.5 to 15.9%) for lumbar spine BMD. In women, the relative contribution of estradiol to BMD variation was even greater than the contribution of body weight.

**Table 3:** Relative contribution of age, body weight, testosterone, and estradiol levels to variation in bone mineral density at the femoral neck and lumbar spine

**Predictor**	**Men**		**Women**	
	**Femoral neck BMD**	**Lumbar spine BMD**	**Femoral neck BMD**	**Lumbar spine BMD**
Age	0.331	0.035	0.279	0.194
	(0.246 – 0.409)	(0.01 – 0.09)	(0.227 – 0.331)	(0.155 – 0.239)
Weight	0.202	0.204	0.065	0.067
	(0.125 – 0.276)	(0.120 – 0.294)	(0.036 – 0.114)	(0.032 – 0.136)
Log (testosterone)	0.003	0.005	0.029	0.031
	(0.001 – 0.044)	(0.001 – 0.056)	(0.1017 – 0.051)	(0.017 – 0.054)
Log (estradiol)	0.025	0.012	0.113	0.117
	(0.004 – 0.078)	(0.001 – 0.058)	(0.081 – 0.153)	(0.085 – 0.159)

Note: The data are shown in percentage and 95% confidence interval in brackets. For example, age accounted for 33.1% of the variation in femoral neck BMD in men. In the presence of age, weight, and testosterone, estradiol further accounted for 2.5% of the variation in femoral neck BMD.

## Discussion

The traditional view of sex hormones and bone health is that estrogens are the main sex steroids affecting bone maturation in women, and that androgens are the corresponding sex steroids in men. However, this view has been challenged by an initial case report [3] and subsequent reports [4,5] which showed that the “female hormone” is actually important for bone mineralization and bone growth in men. However, such a relationship has not been well documented in Asian men, and it is not clear of the magnitude of effect of estrogen in bone mass in men. The present study on Asian men shows that estrogen, not testosterone, was an independent determinant of bone density. However, the contribution of estrogen to the variance of bone density in men is likely to be modest.

Our finding of positive association between estradiol and bone mass in men is largely consistent with previous studies in Caucasian men. Indeed, several observational studies in older Caucasian men have shown that circulating free estrogen or total estradiol, not testosterone, was associated with bone density [18-20] and bone loss [14,21-23]. Our finding is also in line with a recent observation that in Chinese men, low levels of bioavailable E2 were associated with low bone density and greater bone loss [24]. Our results further show that the magnitude of association between estradiol and BMD in men was comparable with or greater than that in women. Collectively, the present data and evidence to date support the conjecture that estradiol is probably more important than testosterone as a determinant of bone growth and bone maintenance in men and women alike.

The mechanism of effect of estrogen on bone is well known. Deficiency of estrogen leads to an increased osteoclastic formation, expanding the remodeling space, increased cortical porosity, and enlarged the resorption area on trabecular surface [25,26]. The net result of these effects is bone loss. Indeed, estrogen deficiency is associated with an increased in bone resorption marker in men [14] and women [27]. Moreover, treatment of elderly men with an aromatase inhibitor resulted in increased bone resorption and decreased bone formation [28]. Treating osteoporotic men with selective estrogen receptor modulator increased femoral neck BMD [29]. Taken together, these studies suggest that estrogen is causally linked to bone mass in men, and that estrogen might be more important for bone health than are declining levels of testosterone.

However, it can not rule out the role of androgens in maintaining skeletal integrity and bone homeostasis in men. Testosterone contributes to the initiation of bone resorption and maintenance of bone formation. Moreover, the majority of estrogens in elderly men is derived from androgens by peripheral conversion [30]. The gene involved in the conversion is the CYP19A1 gene. Men with TTTA repeats in the gene had higher estradiol levels and reduced bone loss than those with lower number of such repeats [31].

Despite the established relationship between estrogen and bone density in men, the magnitude of this association has not been quantified. In this study, by using a sophisticated statistical technique, we were able to estimate that estradiol explained only 1.2 to 2.5% of between-individual variation in BMD. The upper estimate of attributable proportion was ~8%. This relative attribution was much lower than body weight which accounted for 20% of variation in BMD. Most of the association between body weight and BMD is due to muscle mass [32,33]. The dominant contribution of body weight and modest attribution of estrogen imply that in men physical fitness may be more important than sex hormones as a factor for maintaining bone mass in the old age.

In our study, serum levels of testosterone in men remained fairly stable while there was a linear decline of estradiol levels with advancing age in men with modest magnitude of decrease. We found perhaps not surprising that the age-related decrease in estradiol levels in women was greater than that in men, such that before the age of 50, estradiol concentrations in women was higher than that in men, but after the age of 50 years, estradiol levels in women was lower than in men. This finding is consistent with previous studies [21,34-37].

The present results should be interpreted within a number of potential strengths and weaknesses. The study participants were randomly drawn from the general population which should enhance the findings’ external validity. The DXA measurement of BMD is considered gold accurate and reliable, which ensure the study’s internal validity. Moreover, the technique of measurement of estradiol and testosterone was based on the novel Elecsys automated assay, which has been shown to be a precise method for measuring sex hormones over a wide reportable range in serum. Indeed, recent studies have shown that measurement of estradiol and testosterone by this method was highly concordant with the liquid chromatography tandem mass spectrometry methods [38-40]. Nevertheless, we did not measure SHBG, free estradiol and free testosterone in the study. The study was designed as a cross-sectional investigation, and as a result, no causal inferences could be made for the observed relationships between estradiol and bone density. In this study we did not ascertain the phase of menstrual cycle which could affect the measured value of estradiol within an individual. However, the intra-invididual variability was statistically considered as a component of the random error in the multiple linear regression model.

## Conclusion

In summary, these data suggest that estradiol, but not testosterone, was a significant determinant of bone mineral density in Asian men and women. However, the relative contributions of estradiol to the between-individual variation in bone density is likely to be modest for men. This finding is consistent with the hypothesis that estrogen is a key hormone in the skeletal maturation, bone mineralization and maintenance of bone mass in men and women. The finding also provides further supportive evidence for the “unitary model” of pathogenesis of osteoporosis [22].

## Competing interests

The authors declare that they have no competing interests.

## Authors’ contributions

Contributions of the authors to the manuscript included *Study concept and design:* LH-P and TVN; *Acquisition of data:* LH-P. *Analysis and interpretation of data:* LH-P, NDN, and TN; *Drafting the manuscript:* LH-P, TVN and NDN; *Data analysis:* NDN and TVN; *Critical revision of the manuscript:* LH-P, TVN and NDN. All authors read and approved the final manuscript.

## Pre-publication history

The pre-publication history for this paper can be accessed here:

http://www.biomedcentral.com/1471-2474/14/366/prepub
